# An Updated Needs Analysis of Michigan’s Area Agencies on Aging: Supporting Those Who Support Others

**DOI:** 10.1093/geroni/igaf122.3426

**Published:** 2025-12-31

**Authors:** Natalie Delemeester, Joan Ilardo, Angela Zell

**Affiliations:** Michigan State University, Okemos, Michigan, United States; Michigan State University, East Lansing, Michigan, United States; Michigan State University, East Lansing, Michigan, United States

## Abstract

**Background:**

As individuals age, they often develop more complex medical needs that may necessitate assistance with activities of daily living. Such assistance has traditionally been provided by children, and in 2019, there were an estimated 11 million Americans caring for both their own children and their parents. The Michigan Area Agencies on Aging (AAA) are an instrumental part of connecting caregivers with resources such as respite care, caregiver training, and care plan monitoring and development.

**Methods:**

In 2020 and in 2024, a needs assessment of the 16 AAAs in Michigan was conducted via an online questionnaire sent to each AAA director. In 2020, all 16 responded. In 2024, nine responded. The data from the nine from both points in time were then compared.

**Results:**

The responding AAAs prioritized providing respite care and caregiver training in 2020 and 2024. The AAAs reported a 50% decrease of in-person provision of services with a corresponding growth of 125% increase in telephone and use of online service provision. This was in the setting of the elimination of phone and online services from POS contracts. There was also a 228% increase in persons from POS contracts doing caregiver actives who were identified as “other”. All nine AAAs predicted future growth over the next three years.

**Conclusion:**

Understanding the needs of the AAAs is essential for continued governmental planning and distribution of funds. The AAAs anticipate increased future demand and are expanding how they reach caregivers while also utilizing more diverse contracted workers.

